# Protective effects of low dose stabilized haemoglobin solution in experimental global ischaemia-reperfusion injury

**DOI:** 10.1186/2197-425X-3-S1-A912

**Published:** 2015-10-01

**Authors:** L Zacchetti, M Singer

**Affiliations:** Dipartimento di Fisiopatologia Medico-Chirurgica e dei Trapianti, Università degli Studi di Milano, Milano, Italy; Bloomsbury Institute of Intensive Care Medicine, University College London, London, United Kingdom

## Introduction

We previously reported that low doses of the purified stabilized bovine haemoglobin (EXP1) improved survival in a severe haemorrhage reperfusion model with beneficial effects on oxidative stress (unpublished data). We here report additional findings on a similar formulation, EXP2, at different doses using the same model of shock resuscitation.

## Methods

Male Wistar rats (weight 306 ± 15g) underwent arterial and central venous cannulation, tracheostomy, bladder catheterization and placement of a liver tissue pO_2_ sensor (Oxford Optronix, Oxford, UK). Controlled haemorrhage (45% of estimated blood volume) was induced over 15 minutes and sustained shock (MAP target 40-45 mmHg) was maintained for an additional 75 minutes. After crystalloid resuscitation, animals were randomized to receive a bolus injection of either Ringer's lactate (control) or EXP2 at 200, 400 or 900 mg/kg (New A Innovation Limited, Hong Kong). 700 mg/kg is approximately equivalent to a one-unit blood transfusion. Continuous i.v. fluid administration (10 mg/kg/hr) was then infused for the following 4 hours. Echocardiography, blood gas analysis, blood pressure and liver tissue pO_2_ were measured pre- and post- insult, and then at hourly intervals. Two-way ANOVA with post-hoc t testing was used to detect statistically significant differences.

## Results

Four-hour survival was 50%, 87.5%, 66.7% and 62.5% for the control, 200, 400 and 900 mg/kg groups, respectively. Small but significant changes were seen in plasma Hb levels, no changes were seen in lactate or oxygen delivery, while liver tissue pO_2_ increased only in the 400 mg/kg group. Notably, urine output improved at all EXP2 doses, particularly in the 900 mg/kg group, though the pressor effect was identical for all doses of EXP2.

## Conclusions

Low dose stabilized haemoglobin solution has potential utility in the treatment of global ischaemia-reperfusion injury though further work is needed to clarify optimal dose and mechanism(s) of action.

## Grant Acknowledgment

New A Innovation Limited

**Figure 1:**
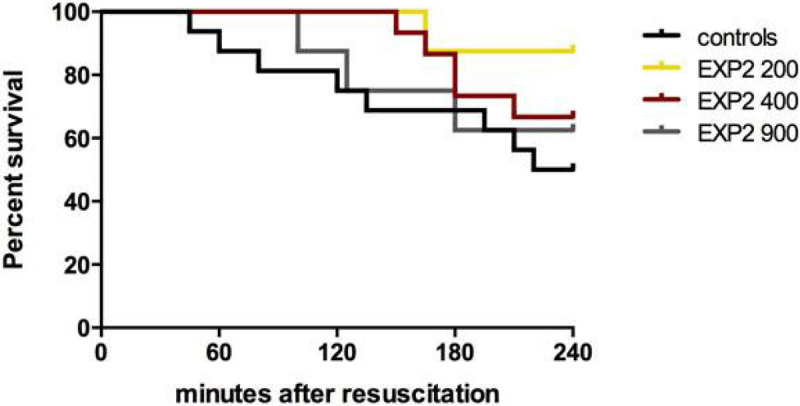
Figure 1

**Figure 2:**
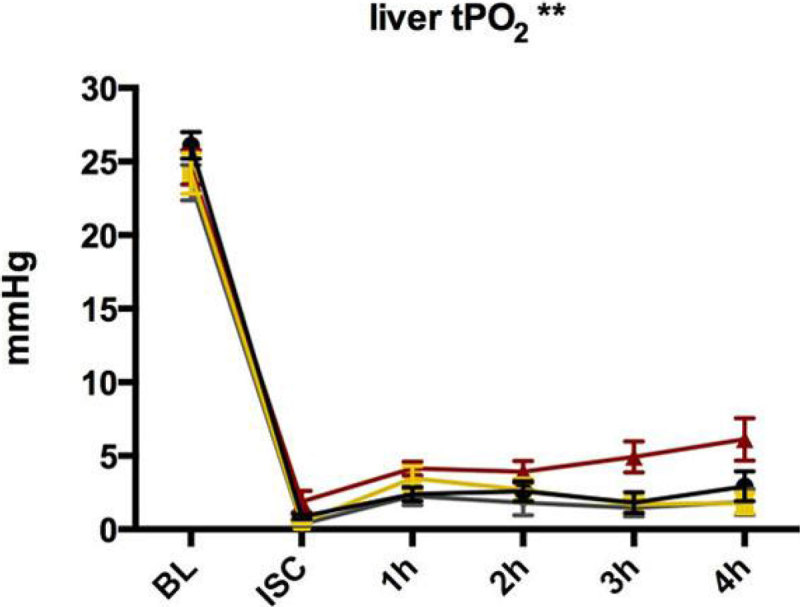
Figure 2

**Figure 3:**
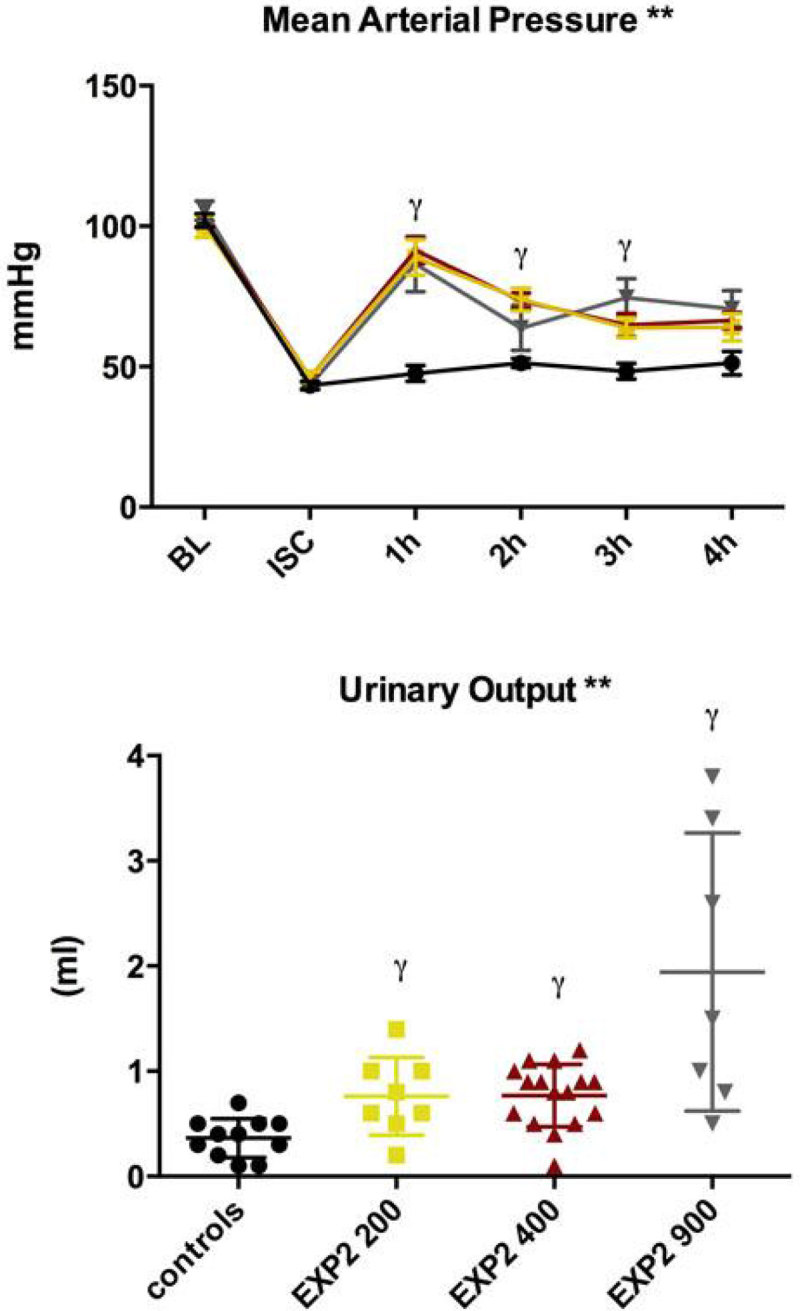
Figure 3

